# Hydrogen-Bond Driven Loop-Closure Kinetics in Unfolded Polypeptide Chains

**DOI:** 10.1371/journal.pcbi.1000645

**Published:** 2010-01-22

**Authors:** Isabella Daidone, Hannes Neuweiler, Sören Doose, Markus Sauer, Jeremy C. Smith

**Affiliations:** 1 Interdisciplinary Center for Scientific Computing, University of Heidelberg, Heidelberg, Germany; 2 Dipartimento di Chimica, Ingegneria Chimica e Materiali, University of L'Aquila, Coppito, Italy; 3 Applied Laser Physics & Laser Spectroscopy, University of Bielefeld, Bielefeld, Germany; 4 Medical Research Council Centre for Protein Engineering, Cambridge, United Kingdom; 5 University of Tennessee/Oak Ridge National Laboratory, Center for Molecular Biophysics, Oak Ridge, Tennessee, United States of America; National Cancer Institute, United States of America and Tel Aviv University, Israel

## Abstract

Characterization of the length dependence of end-to-end loop-closure kinetics in unfolded polypeptide chains provides an understanding of early steps in protein folding. Here, loop-closure in poly-glycine-serine peptides is investigated by combining single-molecule fluorescence spectroscopy with molecular dynamics simulation. For chains containing more than 10 peptide bonds loop-closing rate constants on the 20–100 nanosecond time range exhibit a power-law length dependence. However, this scaling breaks down for shorter peptides, which exhibit slower kinetics arising from a perturbation induced by the dye reporter system used in the experimental setup. The loop-closure kinetics in the longer peptides is found to be determined by the formation of intra-peptide hydrogen bonds and transient *β*-sheet structure, that accelerate the search for contacts among residues distant in sequence relative to the case of a polypeptide chain in which hydrogen bonds cannot form. Hydrogen-bond-driven polypeptide-chain collapse in unfolded peptides under physiological conditions found here is not only consistent with hierarchical models of protein folding, that highlights the importance of secondary structure formation early in the folding process, but is also shown to speed up the search for productive folding events.

## Author Summary

In studies of protein folding evidence exists for early compaction in the unfolded state, although it is unclear whether these compact conformations contain specific secondary structures (through hydrophilic interactions) or whether compaction is a non-specific hydrophobic-driven effect. Here we combine single-molecule fluorescence spectroscopy and molecular dynamics simulation to demonstrate peptide hydrogen-bond-driven polypeptide-chain collapse involving secondary structure formation as the key process in the early stage of folding. Partial structuring in unfolded polypeptide chains is shown to lead to faster contact formation kinetics than would be expected if the unfolded state were populated by featureless random-coils.

## Introduction

The formation of contacts between pairs of residues in an unfolded polypeptide chain is one of the earliest steps in *in vitro* protein folding and is considered to determine the so-called protein folding speed limit [1]. Evidence exists for unfolded states being compact under native conditions [2]–[11], although it is unclear whether these states contain specific secondary structures [5],[9] or whether compaction is a nonspecific hydrophobic-driven effect [10],[12]. The former scenario is consistent with a hierarchical mechanism of folding [13]–[15], in which secondary structures that are local in sequence form first (*e.g.*, the diffusion-collision model [16]). Non-specific compaction is non-hierarchical, requiring condensation for the formation of secondary structures (*e.g.*, the hydrophobic-collapse [12],[17],[18] and nucleation-condensation [19] models). The hierarchical and non-hierarchical models may represent two extremes of a continuum of mechanisms [20],[21], and the position of any given protein on the continuum may depend on, for example, the intrinsic propensity of its amino acid sequence to form secondary-structural elements.

A strategy for obtaining insight into early folding events that is receiving sustained attention from experiment [22]–[30], theory [31]–[36] and atomistic computer simulation [37]–[39] is the study of loop-closure kinetics in unfolded peptides under physiological conditions. The experimental study of loop-closure kinetics requires spectroscopic probes that report on van der Waals contact formation. An effective method is to monitor selective energy transfer such as tryptophan triplet quenching by a cysteine residue [24],[35]. However, since quenching by cysteine at contact is not instantaneous, the quenching rate observed experimentally is less than the rate of actual contact formation (the quenching process is not diffusion-limited, but reaction-controlled [37],[38]). Therefore, extrinsic labels providing faster quenching processes, that have been previously shown to be diffusion limited, can be used [23], [25], [27], [30], [40]–[42]. These labels include oxazine fluorophores that are quenched by tryptophan in single-molecule experiments [30], [40]–[42]. The experiments are amenable to interpretation using atomistic molecular dynamics (MD) simulations since the time scales involved are fast (ns-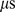), thus allowing bridging of the timescale gap between simulation and experiment that has hitherto existed [43]–[51].

The molecules studied here are glycine-serine (GS) based peptides. Due to their high chain flexibility, their solubility and the absence of a stable folded structure [23],[25],[27],[30],[52] these have been shown to be valuable model systems for studying end-to-end contact formation in “unstructured” polypeptide chains under native conditions, hence providing insight highly relevant to our fundamental understanding of the very first steps of protein folding. Recent FRET experiments and MD simulation have provided evidence for intrachain interactions in these systems in water [28],[39]. However, whether and how the loop closure kinetics is affected by these interactions is still an open question.

Poly-GS peptides exhibit exponential kinetics for end-to-end loop-closure with time constants in the 10–100 nanosecond time range depending on the number of GS units [23],[25],[27],[30]. The end-to-end loop closure rates in peptides with more than 10 peptide bonds exhibit power law scaling as a function of the number of peptide bonds. However, this scaling breaks down for shorter peptides, which exhibit slower rates than obtained by extrapolation of the longer-chain behaviour [25],[27],[30]. The origin of this “rollover” to slower kinetics is unclear. It has been suggested that the rollover is due to the shorter chains being intrinsically stiffer than the longer ones [27],[53], although it has not been ruled out that the rollover might be an artefact due to perturbation by the extrinsic reporter system [33],[35],[36].

Here, a combined experimental and computational study is presented with a twofold aim: understanding the role of intra-peptide hydrogen bonds in the loop-closure kinetics and unveiling the origin of the observed rollover to slower kinetics for the shorter peptides. Loop-closure kinetics in GS peptides of various lengths, labelled with the oxazine derivative MR121 (the fluorescent dye) and tryptophan (the specific quencher) at the terminal ends (MR121-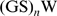, with *n* ranging from 2 to 15), are investigated using fluorescence correlation spectroscopy (FCS) at the single-molecule level with nanosecond time-resolution and MD simulation on the 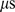 timescale. Excellent agreement is found between the simulated and experimental rate constants, allowing the loop-closure processes to be understood at atomic detail and the role played by intra-peptide hydrogen bonds to be determined.

## Results/Discussion

**Figure 1:**
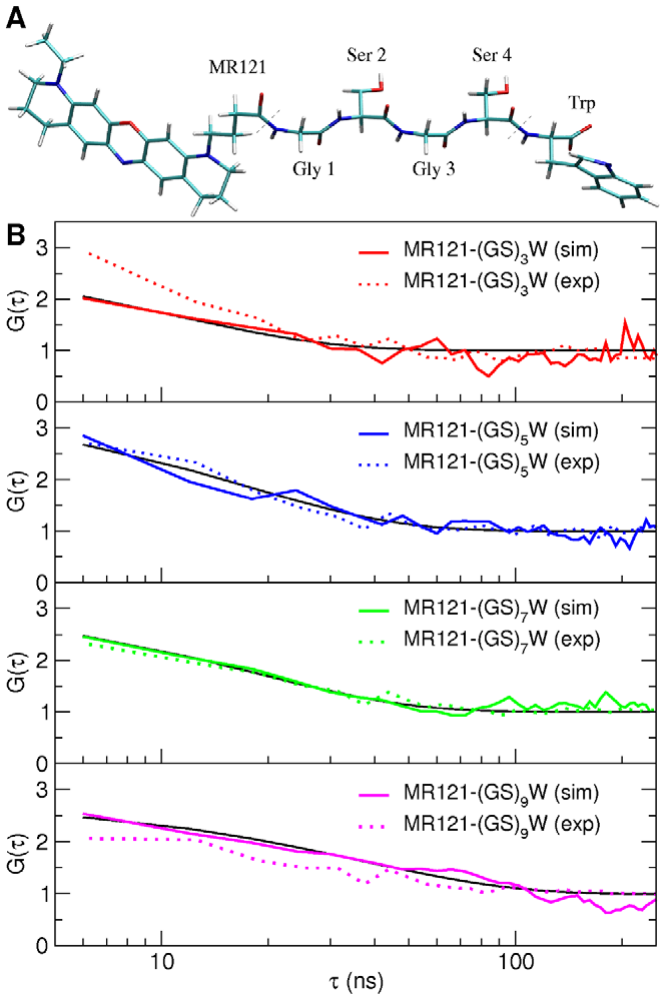
Autocorrelation functions, G(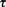), for the labelled peptides. A) Molecular structure of the MR121-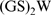 peptide. B) Autocorrelation functions, G(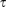) (Eq. 1), calculated from experiment and simulation for four different labelled peptides in the 6–300 ns time range. In black are shown the fits (Eq. 2) to the simulation-derived profiles.

End-to-end contact formation is characterized experimentally here by measuring selective fluorescence quenching of the MR121 dye by a tryptophan residue, the two groups being attached to the opposite termini of a series of poly-GS peptides [30]. The chemical structure of the MR121-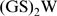 peptide is depicted in Figure 1A. MR121/Trp contact formation and dissociation result in switching the fluorescence “off” and “on”, respectively. The quenching process has been shown in previous work to be diffusion limited [30],[40],[54] and, thus, the underlying kinetics can be revealed by FCS [30].

To aid in interpretation of the experimental results a series of MD simulations of MR121-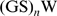 peptides with 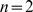, 3, 5, 7 and 9 was performed in explicit solvent at the same conditions as in the experiments, *i.e.*, in aqueous solution at 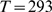 K. Four examples of the second-order autocorrelation functions, 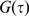, of the fluorescence signal I(t) (Eq. 1) obtained from the experiments and simulations are presented in Figure 1B. The time cut-off is given by the resolution of the FCS experiments (*i.e.*, 6 ns). The profiles are in agreement, justifying the force field and simulation methodology applied.

Assuming a two-state model for the equilibrium between the fluorescent (open) and non-fluorescent (closed) conformational states the time constants (on timescales longer than 6 ns) for end-to-end loop-closure (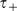) and opening (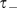) can be calculated from the relaxation time and the amplitude of the exponential function fitted to the data (Eqs. 2 and 3) and are reported in Table 1. Again, 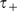 and 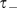 show agreement between simulation and experiment and the 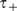 can be compared to previous experiments, showing good agreement [27]. The long-time constants for opening (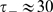
–80 ns) were found to originate from the dissociation of stacked geometries of the aromatic moieties of MR121 and Trp. Assuming that there is no faster process occurring (an assumption imposed by the time resolution of the experiment), the K values yield fractions of open conformations (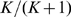) of 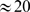
–40% for peptides with 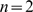
–9, both in experiment and simulation, reflecting the stability of the stacked structures.

**Table 1:** Loop-closure (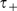) and loop-opening (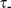) time constants for the labelled peptides

		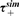	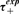	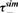	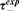
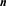	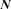	ns	ns	ns	ns
2	4	12.5	14.6(4.5)	26.0	36.7(12.5)
3	6	17.6	17.6(1.5)	31.5	41.7(7.5)
5	10	24.7	20.5(2.1)	53.1	48.5(4.7)
7	14	27.7	32.7(4.5)	52.8	54.3(4.9)
9	18	58.1	48.9(7.5)	81.2	56.5(6.0)
12	24	-	68.4(6.9)	-	73.0(7.1)
15	30	-	102.9(16.5)	-	80.6(10.5)

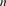
, is the number of glycine-serine units. 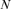, is the number of peptide bonds (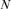 is equal to 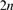). 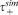, 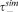 and 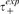, 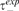 are the time constants derived from a single-exponential fit (Eqs. 2 and 3) of the autocorrelation function G(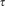) (Eq. 1), over the 6–300 ns time range, for the simulated and experimental labelled peptides, respectively. Errors reported in the experimental values are one standard deviation obtained from three independent measurements.

**Figure 2:**
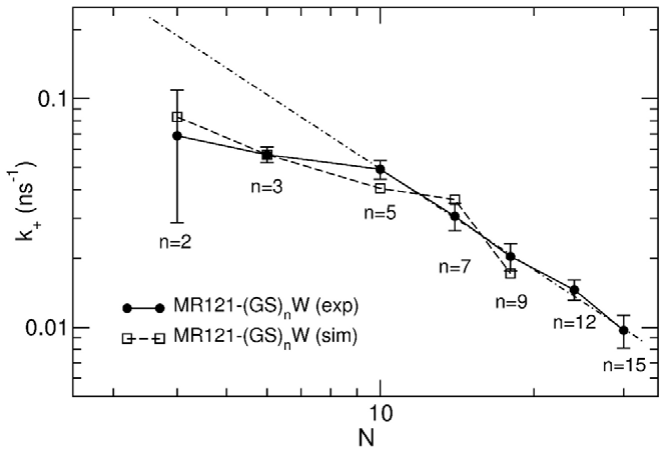
Loop-closure rate constants, 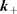, for the labelled peptides. Loop-closure rate constants 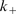 evaluated from experimental- and simulation-derived autocorrelation functions in the 6–300 ns time range for labelled peptides as a function of the number of peptide bonds, 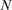. The dotted-dashed line shows the fit of a power-law function with exponent −1.4 to the experimental data for peptides with 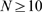.

Concerning the closing rates, experimental [24],[27],[30] and theoretical [31],[53],[55] work has shown that for random-coil chains a scaling law 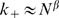 exists for the end-to-end loop-closure rate constants as a function of the number of peptide bonds 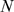, with 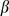 ranging from −1.5 to −2.1 [31],[55]. The 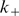 values for the MR121-labelled peptides are reported as a function of 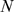 in Figure 2 in a double-logarithmic plot. The rate constants for the MR121-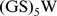 and longer peptides show a power-law dependence resulting in a scaling law 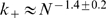, in agreement with the prediction for Gaussian chains. However, for shorter chains, in agreement with previous experimental results [25],[27], the scaling law breaks down. It has been argued that the break-down reflects a pure peptide backbone property, *i.e.*, stiffness of the shorter peptides [27]. However, the breakdown may, in principle, instead arise from perturbation due to the MR121 dye reporter system.

**Figure 3:**
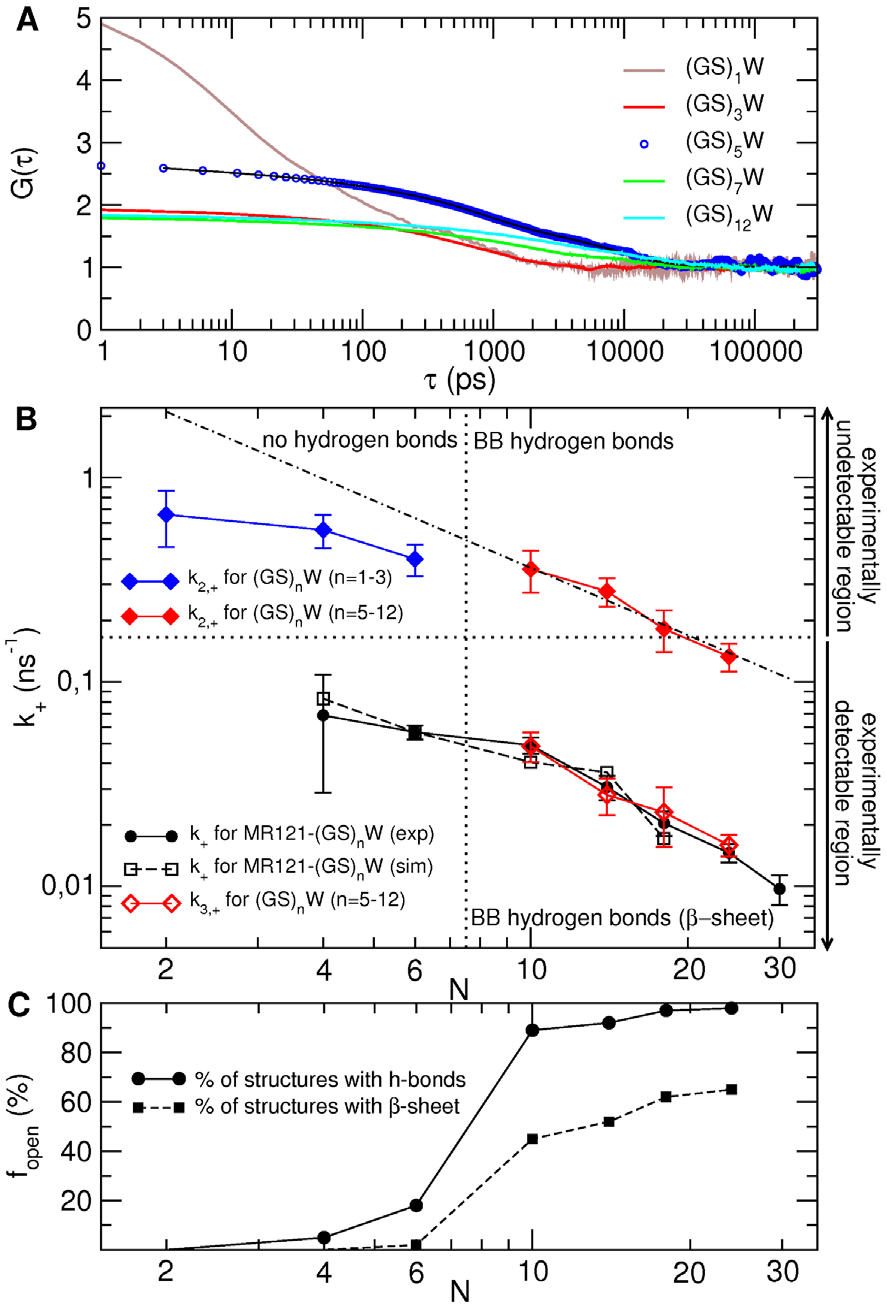
Autocorrelation functions and loop-closure rate constants for the unlabelled peptides. A) Autocorrelation functions, G(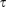), evaluated from simulation for unlabelled peptides with 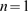, 3, 5, 7 and 12. The curves were fitted as described in the caption of Table 2 (for clarity, the fit is shown only for the 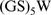 peptide - dashed black line). B) Corresponding loop-closure rate constants, 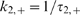 and 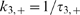, are reported in the upper and lower half, respectively, as a function of the number of peptide bonds, 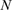. For comparison are shown in black the 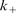 of the labelled peptides evaluated from the experiment (circles) and simulation (open squares). Note that fitting the G(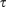) of the unlabelled peptides as in the experiment, *i.e.*, in the 6–300 ns time range, yields 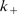 values within the error of the 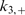 evaluated from the multiexponential fit described in Table 2. The dotted-dashed line shows the fit of a power-law function to the 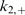 of unlabelled peptides with 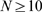. C) Fraction of structures in the open state that possesses peptide hydrogen bonds (solid line) and 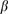-sheet structure (dashed line).

Simulations of the same peptides but without the extrinsic MR121 dye attached to the chain-end allow the origin of the scaling breakdown to be understood. To this end, a series of simulations was performed for 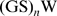 peptides with 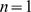, 2, 3, 5, 7, 9 and 12 but without the MR121 dye. Again, for these systems the autocorrelation functions 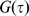 were calculated and analysed. Corresponding time constants, and a description of the fitting procedure used, are reported in Table 2 and 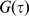 is reported in Figure 3A. The shorter peptides (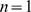
–3) do not show any relaxation process in the experimentally-detectable timescale, *i.e.*, for times longer than 6 ns. Only faster relaxations, 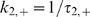, below the experimental time resolution, are present (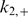 are reported on the upper half of Figure 3B). Peptides with 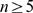 do possess slower closing rate processes, 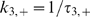, which are within the experimental time resolution and can therefore be compared with experiment (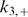 are reported on the lower half on Figure 3B). In addition to this experimentally-detectable relaxation, the longer peptides also possess a faster decay, 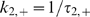 (upper half of Figure 3B).

**Table 2:** Loop-closure (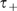) and relaxation (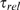) time constants for the unlabelled peptides

	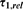		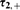		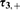	
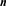	ps	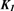	ns	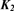	ns	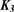
2	57 ( 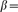 0.6)	26%	1.8(0.2)	60%	-	-
3	102 ( 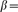 0.7)	26%	2.5(0.6)	64%	-	-
5	34 ( 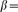 0.7)	16%	2.8(0.7)	35%	20.6(4.0)	37%
7	39 ( 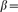 0.8)	13%	3.6(0.6)	35%	35.7(6.5)	40%
9	38 ( 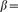 0.6)	16%	5.5(1.1)	40%	43.4(9.5)	40%
12	60 ( 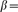 0.7)	17%	7.5(0.9)	35%	62.8(6.5)	43%

The autocorrelation functions G(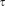) (Eq. 1) of the unlabelled peptides decay by 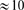
–15% in the first 3 ps, in agreement with femtosecond-timescale spectroscopic data [29] and previous MD results [70]. Beyond 3 ps the curves are fitted using the following function: 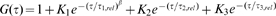. Correlation coefficients were higher than 0.95 and the 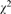 lower than 15. On the 3–500 ps timescale a complex (non-exponential) decay is observed (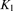, 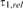), again in agreement with experiment [29], corresponding to a distribution of relaxation times which is found to result from a range of processes, including rotation around single bonds and breaking and forming of intra-chain hydrogen bonds (space restrictions preclude a detailed description of these processes). On the nanosecond timescale one exponential relaxation (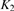, 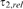) for chains with 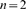 and 3 and two exponential relaxations (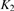, 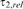 and 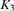, 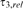) for chains with 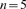, 7, 9 and 12 are observed. 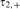 and 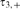 are obtained using Eq. 3. Errors reported in parenthesis are one standard deviation as obtained by dividing each trajectory into two halves.

The 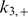 for the longer unlabelled peptides (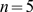, 7, 9, 12) almost coincide with the experimental and simulation-derived 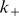 of the labelled peptides (see lower panel on Figure 3B). Therefore, there is no detectable effect of the dye on the closing kinetics of peptides with more than 10 peptide bonds (in the Supplementary Information - Text S1 - evidence of the similarity between the open states in labelled and unlabelled peptides with more than 10 peptide bonds is provided, confirming that the agreement between the corresponding closing rates is not fortuitous). In contrast, however, the labelled peptides with 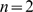, 3 contain an experimentally-detectable slow component both in experiment and simulation, which is absent in the unlabelled 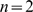, 3 peptides. This is found to arise from open conformations stabilized by hydrogen bonds between the MR121 dye and the backbone (details are given in the Supplementary Information - Text S2). Therefore, the presence of the dye is responsible for the rollover observed for labelled peptides with less than 10 peptide bonds.

The above results do not rule out that the shorter peptides might be intrinsically dynamically different from the longer ones. Indeed, a closer look at the closing processes on the faster, experimentally non-detectable, timescale reveals that the closing rate constants of the longer chains (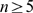) show a power law scaling, but again the scaling breaks down for the shorter peptides with 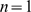, 2 and 3 (upper half of Figure 3B). The rollover to slower closing kinetics in the unlabelled peptides suggests that there is, indeed, an intrinsic effect for peptides with less than 10 peptide bonds.

A structural explanation for the experimentally-detectable closing kinetics and for the differences observed between the short (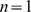, 2, 3) and long (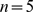, 7, 9, 12) unlabelled peptides (*i.e.*, the absence of 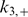 rate constants for the short peptides and the presence of a rollover in the 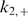) was found in an analysis of the peptide hydrogen bonds. While for the shorter chains (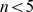) less than 20% of the structures in the open state possesses intra-backbone hydrogen bonds, this value abruptly increases to almost 100% of the structures for peptides with 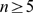 (see Figure 3C). Moreover, 45%, 52%, 62% and 65% of the structures populating the 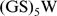, 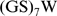, 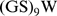 and 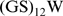 open states, respectively, contain short 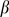-sheet segments, *i.e.*, with two to six consecutive inter-strand hydrogen bonds formed. Hence, in peptides with more than 10 peptide bonds closure occurs from open structures possessing peptide hydrogen bonds, some of which are involved in secondary structure elements, and some not, whereas in peptides with less than 10 peptide bonds, closure occurs from open structures with no hydrogen bonds. The observation of a rollover to slower kinetics and the absence of intra-peptide hydrogen bonds for the shorter unlabelled peptides clearly show that there is an intrinsic stiffness in the short polypeptide chains.

**Figure 4:**
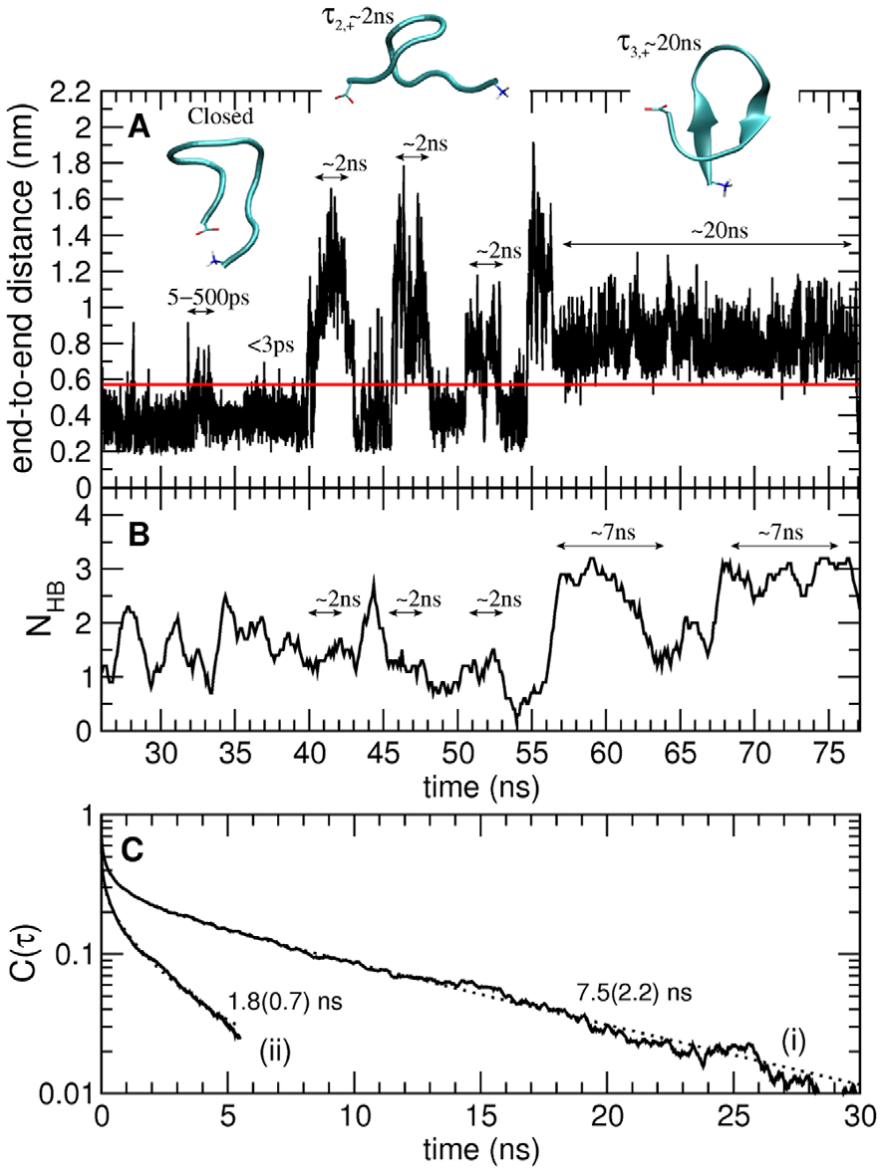
Time-dependent properties evaluated from MD simulation for the 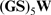 peptide. A) End-to-end minimum distance. The horizontal line indicates the cut-off distance of 0.58 nm used to define if a conformation is closed or open. Loop-closure events on the different timescales can be observed: 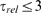 ps; 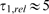
–500 ps; 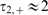 ns; 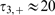 ns. Representative structures of the closed and open states are shown. B) Number of intra-backbone hydrogen bonds. C) Hydrogen bond existence autocorrelation function, 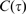, shown for two different kinds of hydrogen bonds present in open configurations: hydrogen bonds involved (i) and not involved (ii) in 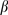-sheet structure. Each of the two 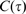 was fitted with a sum of a stretched exponential (in the picosecond time-range) and a single exponential (in the nanosecond time-range). Relaxation times in the nanosecond time-range are taken as the average hydrogen bond lifetimes and are shown in the figure. Errors are indicated in parentheses and correspond to one standard deviation obtained from 2 independent trajectories. Correlation coefficients were higher than 0.99.

Examination of the time dependence of the end-to-end distance and of the number of intra-peptide hydrogen bonds allows the contributions to the end-to-end closing process of the two kinds of peptide hydrogen bonds in open conformations (*i.e.*, those involved in secondary structure formation or not) to be determined. Examples of the time series of the end-to-end distance and of the number of intra-peptide hydrogen bonds for the 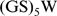 are given in Figure 4A and 4B, respectively - these plots are also representative of the longer peptides. Closing events with 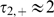 ns occur from structures with one or more peptide hydrogen bonds not involved in secondary structure, while the slower closing processes, with 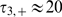 ns, occur from structures with multiple hydrogen bonds involved in short 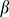-sheet segments (examples of these structures are shown in Figure 4A).

Average lifetimes of the hydrogen bonds were calculated using the hydrogen bond existence autocorrelation function, 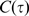, which is the probability that a given hydrogen bond which was intact at the initial time (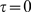) is also found intact at later time, 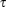 [56]. The 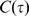 functions were grouped (and averaged) into two groups: one comprising hydrogen bonds involved in secondary structure formation and the other not (examples for the 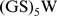 peptide are given in Figure 4C). Both 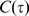 are found to exhibit a fast relaxation on the picosecond timescale that is clearly non-exponential, and a slower, exponential, relaxation. The exponential process has relaxation times of 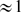
–2 ns for the hydrogen bonds not involved in 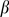-sheet formation, and 7.5 ns, 14.8 ns, 19.5 ns and 22.3 ns for peptides with 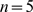, 7, 9 and 12, respectively, for the hydrogen bonds involved in 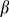-sheet structure.

**Figure 5:**
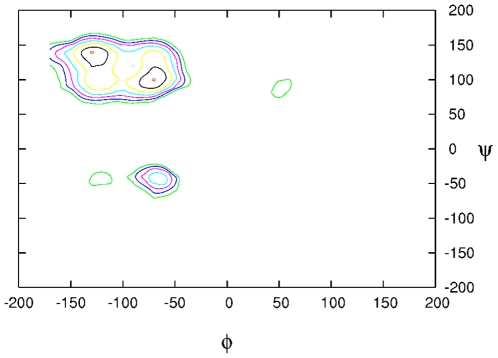
Ramachandran plot of all nonglycine residues evaluated from simulation. Frequency plot, analogous to the Ramachandran plot, showing the 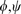 distribution of backbone conformations of all nonglycine residues evaluated from simulation for the 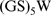 unlabelled peptide. The plot is also representative for the other peptides. The most populated regions are the 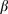-sheet (at around −135Â°;135Â°) and polyproline II, PPII, (at around −75Â°; 140Â°) regions.

The above results clearly show that the experimentally-detectable slow component of the end-to-end closing kinetics found exclusively in the longer peptides arises from the existence in the open state of transient 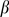-sheet structures, the probability of occurrence and lifetimes of which increases with chain length. Before closing, the chains explore one or a few of these conformations, giving rise to closure dynamics on the 20–100 ns timescale. Interestingly, in the open structures not possessing 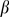-sheet segments, i.e., those from which the faster loop-closure events take place, a common backbone conformation is observed, namely the polyproline II, PPII (see Figure 5). These data confirm previous results [57] showing that the PPII is a dominant conformation in unstructured peptides.

**Figure 6:**
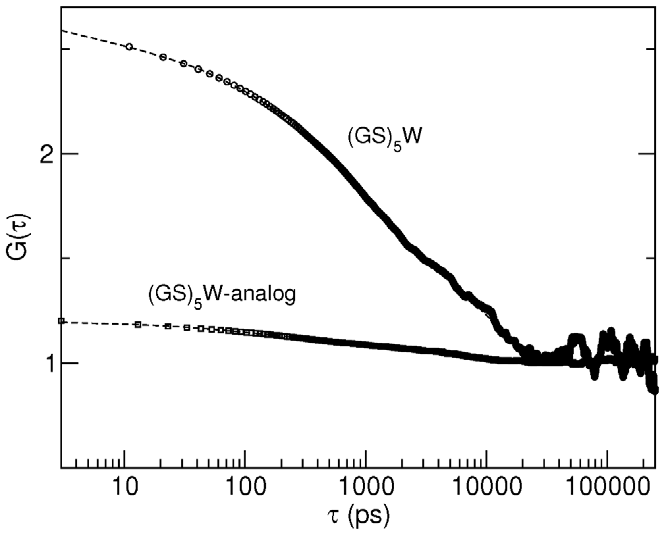
Effect of intra-backbone hydrogen bonds on the loop-closure kinetics. Autocorrelation functions, G(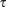), evaluated from simulation for the unlabelled 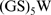 peptide and for a 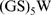-analog that was simulated in the same conditions as the 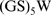 peptide, but with all the charges of the backbone atoms set to zero (except for the two termini). The curves were fitted as described in the caption of Table 2.

Finally, the question arises to whether the presence of transient secondary structures in the unfolded peptides actually accelerates or slows down the loop-closure kinetics relative to the hypothetical system in which no hydrogen bonds, and thus no secondary structure, can be formed. To answer this question a 1.5 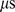 simulation of the 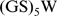 peptide was performed under the same conditions as the previous simulations, but with all the charges of the backbone atoms set to zero (except for the two termini), thus rendering impossible the formation of intra-backbone hydrogen bonds. The end-to-end autocorrelation function G(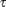) (Eq. 1) was calculated also for this simulation and the resulting average closing times compared with those obtained for the reference simulation (see Figure 6). The average end-to-end loop-closure time in the nanosecond time scale increases by a factor of four (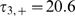 ns vs. 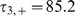 ns) if no hydrogen bonds can be formed. This shows that the formation of hydrogen bonds accelerates the end-to-end loop closure.

**Figure 7:**
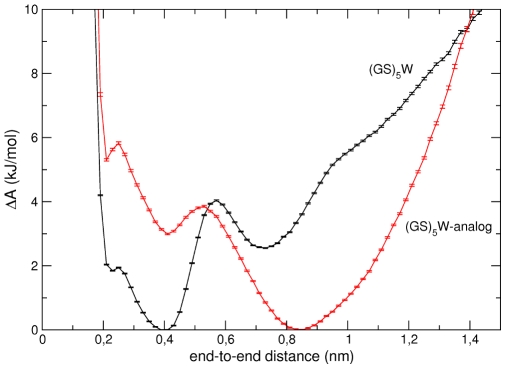
Free energy profiles along the end-to-end distance. Free energy profile along the end-to-end distance calculated from simulation for the unlabelled 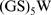 peptide (black) and for the uncharged 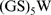-analog (red). The errors bars correspond to one standard deviation obtained from 2 independent trajectories.

To address the possible origin of the acceleration of the closure kinetics by the formation of intra-peptide hydrogen bonds, further analyses were performed. The probability-density-based free energy profile along the end-to-end distance is calculated from simulation for the unlabelled 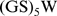 peptide and for the corresponding uncharged-analog (see Figure 7). Both free energy landscapes show two minima, one corresponding to closed conformations (at around 0.4 nm) and the second to open, compact structures (at around 0.7–0.8 nm). The free energy barrier on going from the open- to the closed-state is much smaller for the 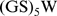 than for the analog, being 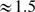 and 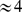 kJ/mol, respectively. A lower barrier leads to faster closing kinetics, as indeed was found here. The effect of hydrogen bond formation on the relative stability of closed and open, compact conformations is, hence, to lower the free energy barrier to closure.

The present results point to a kinetic role played by intra-peptide interactions in driving the end-to-end contact formation in small- to medium -sized polypeptide chains. In previous studies it has been shown that a high percentage of proteins have their N- and C-terminal elements in contact, more than expected on a random probability basis [58]–[60]. A possible rationale for this bias was found in structural and functional aspects, rather than in the kinetics of folding. For example it was suggested that the terminal regions stabilize tertiary and quaternary structure to provide a framework for the active site [58] and that the N-C motif was evolutionarily selected for some functional advantage and is therefore now built into the structural design of many proteins [60]. This can be contrasted with the present work that points to a kinetic role played by intra-peptide interactions in driving the end-to-end contact formation in peptides.

### Conclusions

Understanding the loop-closure dynamics of unfolded peptides provides valuable insight into early steps in *in vitro* protein folding. Here, loop closure of poly-GS peptides is characterized by combining fluorescence correlation spectroscopy with atomistic molecular dynamics simulation.

The experimentally-derived end-to-end loop-closure rate constants are found to decrease with increasing chain length in longer peptides (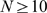), while they become almost independent of chain length for the shorter peptides, as has been previously observed in other experiments that make use of extrinsic probes [25],[27]. Analysis of the simulations reveals that the observed rollover at short chain lengths is due to a perturbation by the extrinsic reporter system. The experimental rate constants of the short chains are found to be determined by transitions to the closed state from open-state conformations containing hydrogen bonds between the MR121 fluorophore and the backbone. However, for peptides with 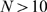 negligible perturbation of the chain dynamics on the experimentally-detectable timescale by the reporter system is seen, as demonstrated by the very good agreement between loop-closure rate constants in peptides with and without the dye reporter system and by the similarity of the corresponding open states.

These results resolve the existing ambiguity regarding the experimentally-determined rollover at short chain lengths in favour of a perturbation effect by the extrinsic reporter systems. Nevertheless, evidence for an intrinsic stiffness of the shorter chains is also provided. The observation of a rollover to slower kinetics and the absence of intra-peptide hydrogen bonds for the shorter unlabelled peptides (*i.e.*, the GS repeats without the extrinsic MR121 dye attached) clearly show that there is, indeed, an intrinsic stiffness in the short polypeptide chains.

The MD simulations allow the dynamical processes driving the end-to-end loop closure to be determined. The nanosecond closing time constants for peptides containing more than 10 peptide bonds correspond to transitions to the closed conformations from open state configurations possessing intra-backbone hydrogen bonds with a broad range of lifetimes. As the chain length increases, the probability of formation of 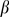-sheet elements increases, leading to the experimentally-detectable length-dependent end-to-end loop-closure time constants on the 20–100 ns timescale, which are determined by the lifetimes of the secondary-structural elements. Early secondary-structure formation in unstructured chains, as found here, is in principle not restricted to 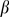-sheet formation and could also, possibly, involve formation of 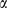-helices, depending on the aminoacid sequence.

The scaling with length of the loop-closure rate constants for chains with more than 10 peptide bonds is found here, as well as in previous studies [24],[25],[27], to be consistent with predictions for Gaussian chains. However, again in line with previous work [34],[39],[61], the presence of partial structuring in unfolded states found here shows that random-coil statistics are not a unique signature of structureless polypeptide chains.

Partial structuring of unfolded states of peptides and proteins has potentially dramatic consequences for the thermodynamics and kinetics of folding [15]. The results presented here reveal structuring in unfolded polypeptide chains driven by backbone hydrogen bonding, also involving transient (of the order of few nanoseconds) 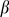-sheet segments. What is most striking, however, is the finding that formation of these peptide hydrogen bonds accelerates end-to-end contact formation by lowering the free energy barrier to closure. In an unfolded polypeptide chain this corresponds to the acceleration of the search for “productive” folding contacts between distant residues. Structuring in poly-GS peptides found here is thus not only consistent with hierarchical models of protein folding, that highlight the importance of secondary structure formation early in the folding process [13]–[16], but is also shown to speed up the search for productive folding events.

## Methods

### MD simulation protocol

MD simulations of a set of MR121-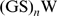 peptides (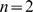, 3, 5, 7 and 9) in water were performed with the GROMACS software package [62] and the GROMOS96 force field [63]. Partial atomic charges for the dye MR121 were taken from Ref. [54]. One peptide molecule was solvated with water and placed in a periodic rhombic dodecahedral box large enough to contain the peptide and at least 1 nm of solvent on all sides at a liquid density of 55.32 mol/l (1 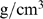) (the starting peptide conformation was taken at the end of a 10 ns-long MD simulation in explicit water in which the peptide was initially modelled in an extended conformation). Water was represented with the simple point charge (SPC) model [64]. Simulations were performed at the experimental temperature of 293 K in the NVT ensemble and isokinetic temperature coupling [65] was used to keep the temperature constant. The bond lengths were fixed [66] and a time step of 2 fs for numerical integration was used.

Periodic boundary conditions were applied to the simulation box and the long-range electrostatic interactions were treated with the Particle Mesh Ewald method [67] using a grid spacing of 0.12 nm combined with a fourth-order B-spline interpolation to compute the potential and forces in between grid points. The real space cut-off distance was set to 0.9 nm. The C-terminal end of the peptides was modeled as 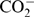 consistent with the experimental pH of 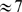 [30]. No counter ions were added since the simulation box was neutral (one positive charge exists on the MR121).

A second series of simulations was performed for the unlabelled 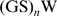 peptides (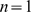, 2, 3, 5, 7, 9 and 12) under the same conditions as the labelled peptides. For these simulations the MR121-dye was replaced by an N-terminal NH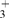 group.

Simulation lengths of the different systems are 1.2, 1.5, 2.5, 3.2 and 3.8 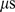 for MR121-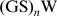 peptides with 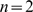, 3, 5, 7 and 9, respectively, and 0.6, 0.8, 1.0, 1.9, 2.5, 3.3 and 4.0 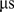 for 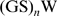 peptides with 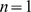, 2, 3, 5, 7, 9 and 12 respectively. The total number of atoms in the simulation box varies between 1366 and 8643, the number of water molecules between 443 and 2821 and the volumes between 13.3 and 84.7 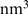 depending on the peptide length.

To test the dependence of the sampled backbone conformations on the force field used, two additional simulations of 500 ns of the 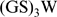 and 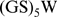 peptides were also performed with a different force field, namely the OPLS-AA [68] force field. Agreement between the two force fields is found in the hydrogen bonding properties, namely 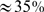
% of the structures populating the open state of the 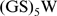 peptide contains short 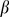-sheet segments, while these are absent in the shorter 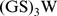 peptide.

Details on the experimental methods, setup and some of the results are reported elsewhere [30].

### Data analysis

The relaxation process of the radiative emission of a fluorescent probe can be analysed via the second-order autocorrelation function of the fluorescence signal I(t) [30]:
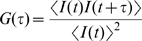
(1)where the angle brackets denote average over all starting times.

Assuming an all-or-none transition between the fluorescent and non-fluorescent states, the following model was used to fit both the experimental- and simulation-derived autocorrelation functions in the labelled peptides:
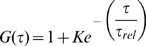
(2)where 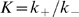 is the equilibrium constant between the open and closed states and 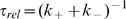 is the mean relaxation time. From 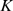 and 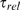, as obtained by Eq. 2 in both the experiments and simulation, average opening, 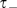, and closing, 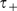, times can be derived as follows:
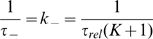


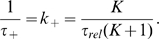
(3)


The criterion for quenching employed in analysing the present simulations is that a non-fluorescent state occurs when the minimum distance between an atom of the conjugated rings of the MR121 and an atom of the rings of the Trp is 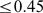 nm (I(t)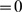) while the state is fluorescent otherwise (I(t)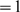). For the non-labelled peptides the minimum distance between the Trp (the rings and the C-terminal 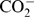 group) and the N-terminal 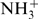 group is taken to define if the chain is in a closed (distance 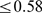 nm) or open (distance 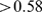 nm) state. The cut-off values (0.45 and 0.58 nm for labelled and unlabelled peptides, respectively) were chosen by constructing for each simulation the probability-density-based free energy profile as a function of the end-to-end minimum distance and by taking as the cut-off value the distance at which the profile exhibits a free energy barrier for escaping from the global minimum at short distances (a representative example is given in Figure 7).

For the definition of a 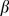-sheet segment the program Dictionary of Protein Secondary Structure (DSSP) with the default hydrogen-bond cutoff parameter of 0.5 kcal 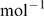 was used [69].

## Supporting Information

Text S1 Characterization of the open state in labelled and unlabelled peptides.
(0.10 MB PDF)

Text S2 Effects of the extrinsic probe on the chain dynamics.
(0.47 MB PDF)
