# Supplementary material for: Hydrogen-Bond Driven Loop-Closure Kinetics in Unfolded Polypeptide Chains
Source: PLoS Comput Biol. 2010 Jan 22;6(1):e1000645. doi: 10.1371/journal.pcbi.1000645 (PMC2799665; doi:10.1371/journal.pcbi.1000645)
Supplement: Text S1 — Characterization of the open state in labelled and unlabelled peptides. (0.10 MB PDF) [file pcbi.1000645.s001.pdf]

Supporting Information

Text S1

Characterization of the open state in labelled and unlabelled peptides.

The open states in the longer peptides, *i.e.*, with more than 10 peptide bonds, are hereby analyzed to assess their similarity in labelled and unlabelled peptides. To structurally characterize the open states the fraction of configurations possessing peptide hydrogen bonds and the fraction possessing transient β-sheet segments was calculated in each open state and is reported in Fig. S1. The two ensembles are similar, with the open state of the labelled peptides showing a slightly lower content of secondary structure.

The dynamical properties of the open states are assessed by evaluating the average lifetimes of the peptide hydrogen bonds (the analysis is the same as described for the unlabelled peptides in the main text of the manuscript and in Fig. 4 therein). In Fig. S2 the hydrogen bond existence autocorrelation function, C(τ), of the hydrogen bonds involved in β-sheet structure is shown for the labelled and the unlabelled peptide with n=9 (the comparison is also representative for the other peptides with more than 10 peptide bonds). The average lifetimes in the nanosecond time range are also reported in the figure and are in agreement within the statistical error.

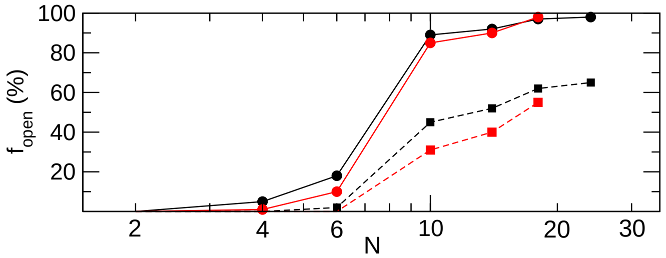

Figure S 1: Fraction of structures in the open state possessing peptide hydrogen bonds (solid line) and β-sheet structure (dashed line) in the unlabelled (black) and labelled (red) peptides. The black curves are the same as those presented in the manuscript in Fig. 3c.

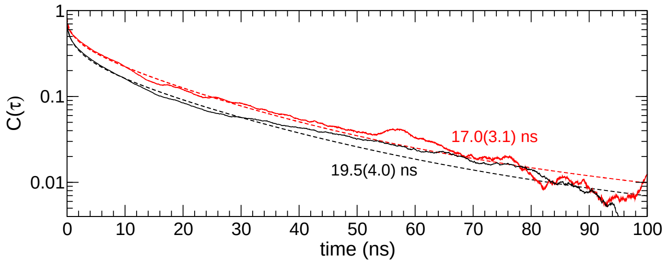

Figure S 2: Hydrogen bond existence autocorrelation function, $C(\tau)$, shown for hydrogen bonds involved in β-sheet structure that are present in the open states of the unlabelled (GS)$_9$W peptide (black) and the labelled MR121-(GS)$_9$W peptide (red). Each $C(\tau)$ was fitted with a sum of a stretched exponential (in the picosecond time-range) and a single exponential (in the nanosecond time-range). Relaxation times in the nanosecond time-range are taken as the average hydrogen bond lifetimes and are shown in the figure. Errors are indicated in parentheses and correspond to one standard deviation obtained by dividing each trajectory into two halves. Correlation coefficients were higher than 0.99.
