# Supplementary material for: Hydrogen-Bond Driven Loop-Closure Kinetics in Unfolded Polypeptide Chains
Source: PLoS Comput Biol. 2010 Jan 22;6(1):e1000645. doi: 10.1371/journal.pcbi.1000645 (PMC2799665; doi:10.1371/journal.pcbi.1000645)
Supplement: Text S2 — Effects of the extrinsic probe on the chain dynamics. (0.47 MB PDF) [file pcbi.1000645.s002.pdf]

Supporting Information

Text S2

Effects of the extrinsic probe on the chain dynamics.

The long closing times ($\tau_+ \approx 15$ ns) observed in the short labelled peptides (n=2, 3), which are instead absent in the corresponding unlabelled peptides, are found to arise from transitions to the closed state from open conformations stabilized by hydrogen bonds between the MR121 dye and the polypeptide chain, the average lifetime of which is of the order of 10 ns, as calculated from the corresponding hydrogen-bond existence autocorrelation function. An example is given in Fig. S1 for the MR121-$(GS)_3$W peptide. Such hydrogen bonds cannot form in the unlabelled peptides, in which, indeed, closing processes of $\approx$15 ns are not present.

Hydrogen bonds between the MR121 dye and the chain can also form in the longer labelled peptides (n=5, 7 and 9), with lifetimes up to tens of nanoseconds, hence contributing to some extent to the slow closing processes. However, as seen for the unlabelled peptides, the slow end-to-end closing times on the 20-100 ns timescale mainly arise from transitions to the closed state from open conformations possessing β-sheet segments (results are very similar as shown in the manuscript for the unlabelled peptides). The relative abundance in the open state of these two kinds of hydrogen bonds (β-sheet and MR121-backbone) was calculated for all peptides and is reported in Fig. S2. The hydrogen bonds involving only the MR121 dye contribute $\approx$100% in the shortest peptides (n=2), while they contribute $\approx$10% in the longer peptide (n=9). Hence, MR121-backbone hydrogen bonds do not contribute significantly to the end-to-end contact formation rates in the longer peptides, as also confirmed by the agreement between labelled and unlabelled closing rates shown in Fig. 3b of the main text. The reporter system, thus, does not perturb the loop closure rates of peptides with more than 10 peptide bonds.

In addition to the physical perturbation on the short labelled peptides described above caused by the extrinsic reporter system, another kind of perturbation is also observed, *i.e.*, the dye-Trp contact formation does not always report on backbone closing processes for the shorter chains. This is shown by the construction of the two-dimensional free energy maps as a function of the MR121-Trp minimum distance and the actual backbone end-to-end distance, *i.e.*, the distance between the N-terminal, N_t, and C-terminal, C_t, atoms (see Fig. S3). Analysis of the maps shows that the backbones of the shorter peptides (n=2, 3) have higher probabilities of being almost extended (N_t-$C_t \approx 1.1$ nm), than closed (N_t-$C_t \approx 0.3$ nm), in conformations in which the probe, MR121, and the quencher, Trp, nevertheless form stacked complexes (see structure (a) in Fig. S3) and are thus detected as

being “closed” in the experiment. In contrast, in the longer chains (n$\geq$5) non-fluorescent closed conformations also have the ends of the backbone in contact (N_t-$C_t$$\approx$0.3 nm). This shows that, for the shorter chains, fluorescent and non-fluorescent conformations do not always correspond to open and closed backbone conformations, respectively.

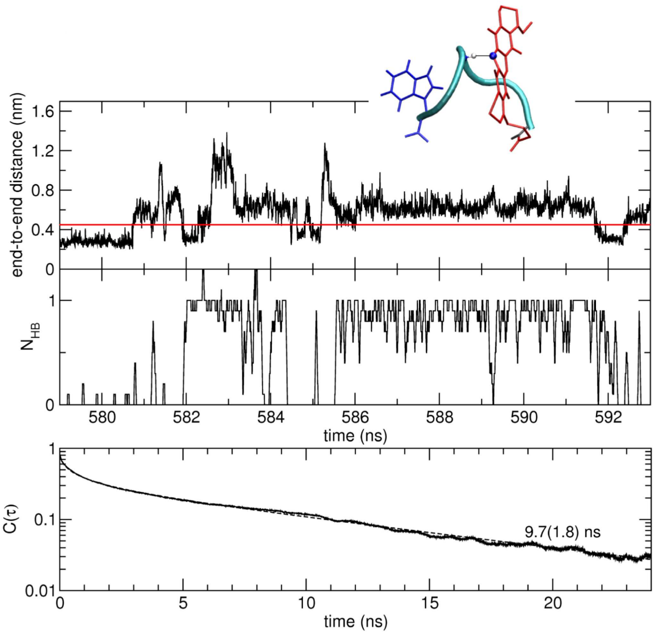

Figure S 1: Time-dependent properties evaluated from MD simulation for the MR121-(GS)3W peptide. Top: MR121-Trp minimum distance. The red horizontal line indicates the cut-off distance of 0.45 nm used to define if a conformation is closed or open. A representative structure of an open conformation with a hydrogen bond between the MR121 and the polypeptide chain is shown. Middle: Number of MR121-backbone hydrogen bonds. Bottom: MR121-backbone hydrogen bond existence autocorrelation function, $C(\tau)$. $C(\tau)$ was fitted with a sum of a stretched exponential (in the picosecond time-range) and a single exponential (in the nanosecond time-range). The relaxation time in the nanosecond time-range is taken as the average hydrogen bond lifetime and is shown in the figure.

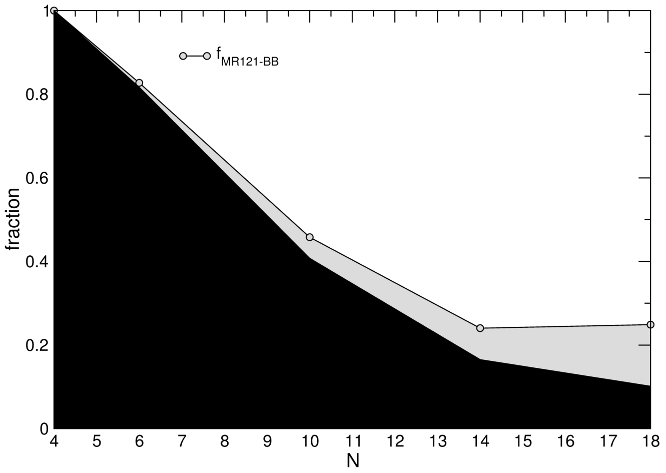

Figure S 2: Given the total number of long-lived hydrogen bonds in the open state of the labelled peptides, $N_{long} = N_\beta + N_{MR121-BB}$, where N_β is the number of hydrogen bonds involved in β-sheet formation and $N_{MR121-BB}$ is the number of hydrogen bonds between the MR121 dye and the backbone, the relative abundance of dye-backbone hydrogen bonds, $f_{MR121-BB} = N_{MR121-BB}/N_{long}$, is calculated and is shown in grey. In black is shown the fraction of the long-lived hydrogen bonds that are formed between the dye and the backbone and that are present alone, *i.e.*, no β-sheet hydrogen bonds are formed at the same time in the given structure. N is the number of peptide bonds.

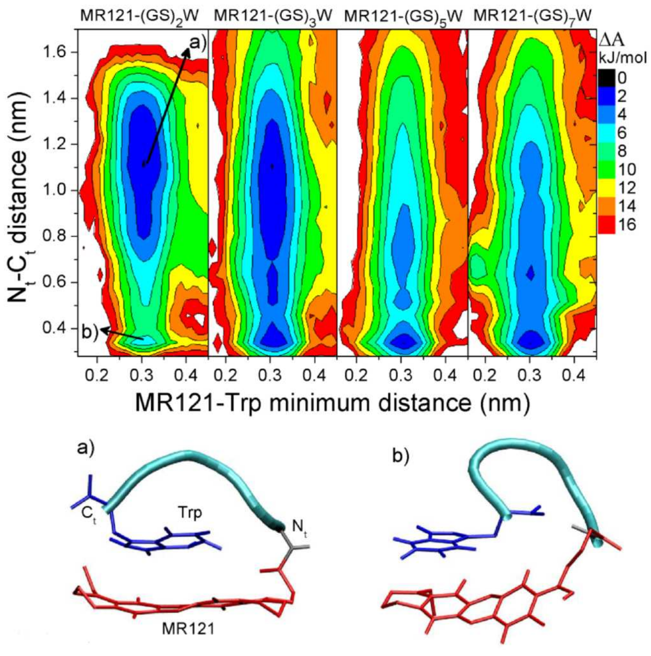

Figure S 3: Two-dimensional free energy maps as a function of the MR121-Trp minimum distance and the N- and C-terminal (N$_t$-C$_t$) distance, evaluated from MD simulation as $\Delta A = -RT \ln \rho_{eq}/\rho_{eq}^{ref}$, with ρ_{eq} the corresponding equilibrium probability density. The state with the highest probability density, ρ_{eq}^{ref}, is taken as the reference state. Representative structures of minima (a) and (b) are shown for the MR121-(GS)$_2$W peptide.
